# Extrapulmonary Tuberculosis: *Mycobacterium tuberculosis* Strains and Host Risk Factors in a Large Urban Setting in Brazil

**DOI:** 10.1371/journal.pone.0074517

**Published:** 2013-10-02

**Authors:** Teresa Gomes, Solange Alves Vinhas, Bárbara Reis-Santos, Moisés Palaci, Renata Lyrio Peres, Paola P. Aguiar, Fabiola Karla Correa Ribeiro, Hebert Silva Marques, Valdério do Valle Dettoni, John L. Johnson, Lee W. Riley, Ethel Leonor Maciel

**Affiliations:** 1 Graduate Program in Infectious Diseases, Federal University of Espírito Santo, Vitória, Brazil; 2 Tuberculosis Control Program, Federal University of Espírito Santo, Vitória, Brazil; 3 Tuberculosis Research Unit, Department of Medicine, Division of Infectious Diseases, Case Western Reserve University, Cleveland, Ohio, United States of America; 4 Division of Infectious Disease and Vaccinology, School of Public Health, University of California, Berkeley, California, United States of America; 5 Graduate Program in Public Health, Federal University of Espírito Santo, Vitória, Brazil; St. Petersburg Pasteur Institute, Russian Federation

## Abstract

**Background:**

Factors related to the development of extrapulmonary forms of tuberculosis (EPTB) are still poorly understood, particularly in high-endemic countries like Brazil. The objective of the paper is to determine host and *Mycobacterium tuberculosis* (MTB) strain-related factors associated with the development of EPTB in Espírito Santo state, Brazil.

**Methods and Findings:**

We conducted a retrospective laboratory-based surveillance study of new tuberculosis (TB) cases diagnosed in Espírito Santo state, Brazil between 1998 and 2007. We genotyped 612 isolates of MTB from 606 TB patients using spoligotyping and *IS6110*-restriction fragment length polymorphism (RFLP) typing and compared sociodemographic and clinical characteristics of patients with pulmonary TB (PTB) and EPTB. Among 606 patients, 464 (77%) had PTB, 79 (13%) had EPTB, 51 (8%) had both, and 12 (2%) had miliary TB. The *IS6110* RFLP analysis demonstrated that 250 (41%) isolates belonged to clustered RFLP patterns, 27 (11%) of which were from EPTB. We identified 73 clusters including 35 (48%) composed of 2 isolates each. By spoligotyping, 506 (83%) MTB isolates fell into known patterns and 106 (17%) fell into patterns with no family assignment; 297 (48%) isolates belonged to the Latin-American Mediterranean family. Higher school level (4-7 years OR: 0.16 95% CI 0.34-0.73 and > 8 years of education, OR 0.06 95% CI 0.009-0.50) white ethnicity (OR: 2.54 95% CI 1.03-6.25) and HIV infection (OR: 16.83 95% CI 5.23-54.18) were associated with EPTB. No specific strain lineage or percentage of clustering was associated with EPTB.

**Conclusions:**

These results demonstrate that risk factors for EPTB are related more to host than to MTB strain lineage characteristics.

## Introduction

Tuberculosis (TB) is the second leading cause of death due to infectious disease worldwide [1]. In 2010 6.2 million new TB cases occurred globally including 800,000 (13%) patients with extrapulmonary tuberculosis (EPTB) [1]. In 2010, Brazil reported 81,946 new cases of TB of which 12% were EPTB [2].

Reported host risk factors for EPTB include HIV (human immunodeficiency virus) infection, younger age, female sex and non-white race [3-5]. In contrast, little is known about bacterial determinants of the clinical site of TB. Molecular epidemiologic studies of *Mycobacterium tuberculosis* (MTB) complex have been done to try to understand whether pathogen factors such as phylogenetic lineage account for differences in clinical sites of TB [6-8]. Pathogen characteristics such as genetic expression of virulence factors or the ability to evade host immune defences or genetic, cultural and environment of the host may determine whether patients present with extrapulmonary as opposed to pulmonary TB [9-11].

In a recent study from the United States (US), Indo-Oceanic and East Indian phylogenetic lineages of the infecting MTB strain were reported to be associated with a higher proportion of exclusively EPTB, even when controlled for region of birth, race/ethnicity, HIV infection status and age [12]. In contrast, in another study from India, there was no association between mycobacterial lineage and EPTB [13].

Espírito Santo (ES), Rio de Janeiro, São Paulo and Minas Gerais states that form the southeast region of Brazil report the highest number of TB cases in Brazil [14]. ES State has a population of 3.5 million persons and a TB incidence of 36.7 per 100,000 habitants in 2010. In the same year, 7.4% of patients with newly diagnosed TB were HIV-infected [15,16].

To further characterize the relative importance of host versus strain factors in determining TB clinical manifestation in another region of the world, we conducted a retrospective laboratory study in ES State, Brazil.

## Methods

### Ethics Statement

This study was part of a larger project of our laboratory that was approved by the Institutional Review Board of the Federal University of Espirito Santo, Brazil, under number 121/06.

Sputum cultures for persons evaluated for suspected TB by clinics in the Vitória metropolitan area are done routinely by the TB Reference Laboratory located at the Infectious Diseases Laboratory of the Federal University of Espirito Santo (UFES). *M. tuberculosis* isolates from positive cultures are routinely stored by this laboratory for use in outbreak investigations and epidemiologic surveillance. This study was a retrospective analysis of data collected routinely during activities of the state TB control program. No patients were contacted to request additional information. The study was reviewed and approved by the institutional review board of UFES who granted permission for use of the MTB isolates and clinical data for the purposes of the study and waived the need for written informed consent from participants as the study involved no more than minimal risk and was done with existing microbiology specimens. All patients had an identification number for clinical purposes. All cultures had a different accession number for laboratory purposes. To protect patient confidentiality, only one investigator (ELM) had access to both de-identification codes; she performed the linkage of the clinical and culture databases for this study. After linkage, a new code number was created for each record for use in the study analysis.

### Study Population

This was a retrospective laboratory-based surveillance study of new TB cases diagnosed in ES State, Brazil between 1998 and 2007 at the state TB reference laboratory in the Núcleo de Doenças Infecciosas at the Federal University of ES. We analyzed stored isolates obtained from all culture-confirmed patients with TB diagnosed during this period. All cultures done at the reference laboratory were included.

Although cultures were performed on different clinical samples depending on the patient’s presenting signs and symptoms, most were done on deep respiratory specimens, mainly sputum with a lesser number of bronchial and gastric lavage specimens. Samples examined for suspected EPTB included aspirates and biopsies of lymph nodes and other sites and urine, peritoneal fluid, pleural fluid, cerebrospinal fluid, pericardial fluid and blood cultures.

Resource-constrained, high TB burden countries like Brazil are unable to afford mycobacterial cultures for all TB suspects. Cultures are recommended for special circumstances such as clinically suspected TB in patients with acid fast bacillus (AFB) negative smears, persons with radiographic findings suspicious for TB, retreatment cases, evaluation of HIV-infected individuals with suspected TB, suspected cases of drug-resistant TB, EPTB, and TB in vulnerable populations (prison inmates, nursing home patients, homeless individuals and health care workers) [17]. We therefore included only isolates from suspected TB cases where cultures were performed.

Clinical and epidemiologic characteristics of the patients were abstracted from laboratory records, medical files, and the Brazilian National Surveillance System (SINAN), and were categorized as: age in years (0 - 9; 10-19, 20-39; 40-60; >60), gender (male/female), ethnicity (white, non-white, not reported), educational level completed (illiterate, 0 - 3 years, 3-7 years; > 8 years, not reported), AFB smear (positive, negative, not done) and HIV status (positive, negative, not done). Patients with information “not reported” or “not done” were included in the descriptive analysis, but were excluded from the comparative analysis. HIV status is recorded in SINAN based on a laboratory test result, not self-report. Some patients in this studies who did not have an HIV status recorded in SINAN had their HIV status entered locally based on local laboratory test results.

Developed nations recommend performing drug susceptibility testing (DST) for all patients at the time of TB diagnosis [18]. Countries with limited resources, such as Brazil, do not follow this practice and DST is only recommended for special cases, such as retreatment after failure, relapse, patients with suspected primary resistance and case contacts of resistant tuberculosis [19]. In this study, we have only reported the DST results from isolates of patients who had DST requested.

The site of disease was assigned according to standard surveillance classification definitions [1,20,21]. Cases were classified as having exclusively pulmonary (PTB), both pulmonary and extrapulmonary (PTB+EPTB), miliary (disseminated) and exclusively extrapulmonary (EPTB) TB. EPTB was further stratified into 7 categories: pleural, peripheral lymphatic, genitourinary, bone and joint, ocular, meningeal, and others.

### IS*6110* Restriction Fragment Length Polymorphism Analysis

Genomic DNA isolation and P*vu*II- IS*6110* restriction length polymorphism (RFLP) analysis was performed according to standard methods [22].

The IS*6110* RFLP band patterns were analyzed by the BioNumerics software version 6.5 (Applied Maths, Sint-Martens-Latem, Belgium). A dendrogram was constructed to show the degree of similarity among the isolates by un-weighted pair group method of arithmetic average (UPGMA) and the Dice index (1.0% tolerance, 1.5% optimization). Two or more strains with indistinguishable RFLP patterns (fingerprint) were defined as belonging to the same RFLP cluster. As described in other studies, strains belonging to a cluster group were considered to represent TB cases resulting from recent infections, while unique RFLP pattern strains were considered to represent reactivation TB cases from infections acquired in the remote past [23,24]. Strains with RFLP patterns shown to be at least 70% similar were defined as belonging to the same family [25].

### Spoligotyping

All MTB isolates were spoligotyped by a commercial kit (Ocimum Biosolutions Ltd., Hyderabad, India) according to the manufacturer’s protocol [26,27]. The results were recorded in a 43-digit binary format representing the 43 spacers. The spoligotyping patterns were compared with an updated SpolDB4database - SITVIT WEB of Pasteur Institute of Guadeloupe (http//:www.pasteur-guadeloupe.fr: 8081/SITVIT_ONLINE) that provides information on MTB spoligotypes [27,28].

### Long Sequence Polymorphism (LSP)

A multiplex PCR (Polymerase Chain Reaction) adapted from Gibson et al. was performed to differentiate isolates belonging to the RDRio lineage [29]. The PCR reaction was performed in a final volume of 25 µL, containing 20pmol of primers BridgeF: 5 ‘- CAC TCC GGC TGC CAA TCT CGT C -3', BridgeR: 5 ‘- CAC CGC GAG GCT GAA TGA GAC CA -3', IS1561F: 5 ‘- GAC CTG ACG CCG CTG ACA C -3', IS1561R: 5 ‘- CAC CTA CAC CGC TTC CTG CC -3'; 1U Taq polymerase (Invitrogen Life Technologies, USA), buffer 1X, MgCl_2_ MgCl_2_ 2.0 mM, DMSO 5%, dNTP 0.2 mM deionized water and 20ng of genomic DNA. The amplification was done in a Gene Amp PCR System 2400 thermocycler (Perkin Elmer, USA). The cycle conditions were 95°C for 10 min, followed by 35 cycles at 95°C for 1min, 60°C for 1min and 72°C for 4 min, and a final extension at 72°C for 10 min. The PCR products were detected in 1.5% agarose gel treated with ethidium bromide, under UV transillumination. The identification of RDRio or non-RDRio strain genotypes was established according to a PCR product band size; the presence of a band of 1175 bp indicated RDRio and a band of 530-bp indicated non-RDRio strains.

### Statistical Analysis

A descriptive analysis of genotyping and epidemiologic data was done followed by comparison of patients with exclusively EPTB and PTB. Covariates used included socio-demographic (age, ethinicity, gender, education), clinical (HIV status and sputum AFB smear positivity) and genotypic [percentage of clustering by RFLP, if LAM (Latin- American- Mediterranean) family or not, and presence or absence of RDRio sub lineage] factors. A previous report from Rio de Janeiro, a neighbouring state of ES, suggested that the sublineage RDRio is linked to higher virulence and greater weight loss [25], so this covariate was included in our analysis.

Regarding the site of TB disease, spoligotying families and TB clinical sites were compared by chi-squared test. All other covariates were compared by chi-square test and logistic regression, estimating crude odds ratio (OR) and adjusted OR, respectively including 95% confidences interval (CI) for both. The choice of only the variable "family or not LAM" entered the logistic regression in contrast to other families, since this is the most common spoligotype family in Brazil [28].

Patients with both PTB and EPTB and miliary TB cases were excluded from the primary comparison due to difficulty in classifying them. Secondary analyses were done for (a) patients with both PTB and EPTB versus PTB; and (b) both PTB and EPTB versus EPTB.

## Results

### TB Clinical Sites

Five thousand four hundred and eighty-one cases of TB cases were notified to the TB program in metropolitan Espirito Santo state during the study period. Eight hundred and twenty-four (15%) had culture performed and 606 (11%) of these patients had complete genotyping data and were included in this study.

**Figure 1:**
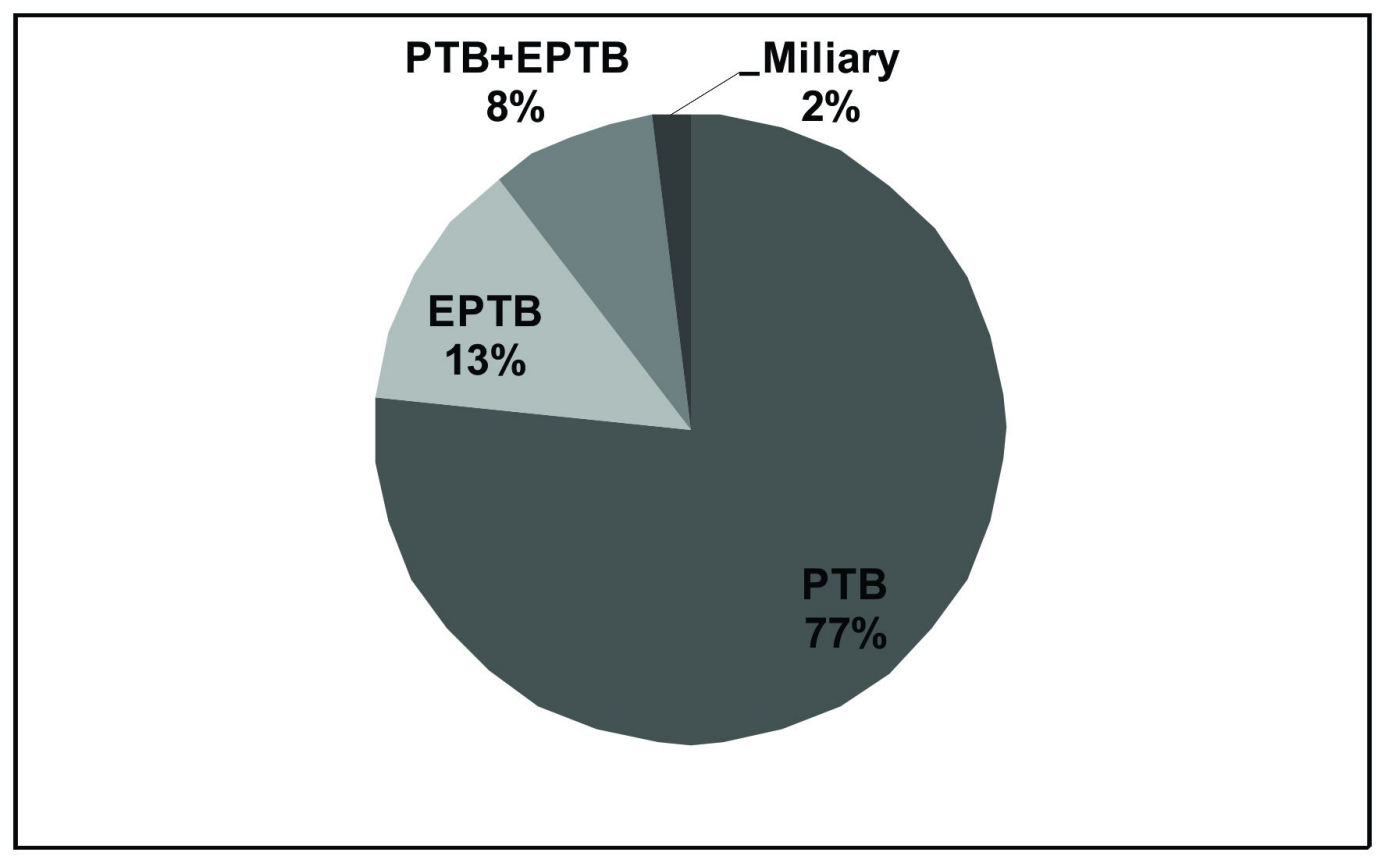
Tuberculosis sites of disease, Espírito Santo State, Brazil.

Of the 606 TB patients in our study population, we found 464 (77%) PTB cases, 79 (13%) EPTB cases, 51 (8%) with both PTB + EPTB, and 12 (2%) with miliary TB (Figure 1). From 130 EPTB and PTB+EPTB cases, 55 (42.3%) isolates were from peripheral lymph nodes, 36 (27.7%) were from pleural fluid, 13 (10%) were from the genitourinary tract, 4 (3%) were from bone and joints, 7 (5.4%) were from cerebral spinal fluid, and 15 (11.6%) were from other sites. The EPTB clinical sites were compared by RFLP cluster, and no meaningful distinction was found (p = 0.57, Chi square test) (Table 1).

**Table 1:** Distribution of *IS6110* RFLP clustered vs unclustered strains according to different sites of infection in TB patients in Espirito Santo state, Brazil, 1998-2007.

	**RFLP - Clusters**		
**Site of infection**	Clustered (%)	Unclustered (%)	**Total**
**Extrapulmonary TB**	27 (24.0)	52 (66.0)	79 (100)
peripheral lymph nodes	21 (38.2)	34 (61.8)	55 (100)
pleural	11 (30.6)	25 (69.4)	36 (100)
genitourinary	5 (38.5)	8 (61.5)	13 (100)
bone and joints	2 (50.0)	2 (50.0)	4 (100)
meningeal	5 (71.4)	2 (28.6)	7 (100)
others	7 (46.7)	8 (53.3)	15 (100)
Total (Extrapulmonary TB)	51 (39.2)	79 (60.8)	130 (100)
**Pulmonary TB**	196 (42.0)	268 (58.0)	464 (100)

P value: 0.57

TB: Tuberculosis. RFLP : Restriction Fragment Length Polymorphism.

### MTB Genotypes

Good quality RFLP patterns and spoligotyping data were obtained from 612 isolates from 606 patients; the greater number of isolates than patients is due to the fact that 6 patients with EPTB+PTB had isolates with different RFLP patterns from PTB and EPTB sites and both isolates were therefore included in the genotype analysis.

**Figure 2:**
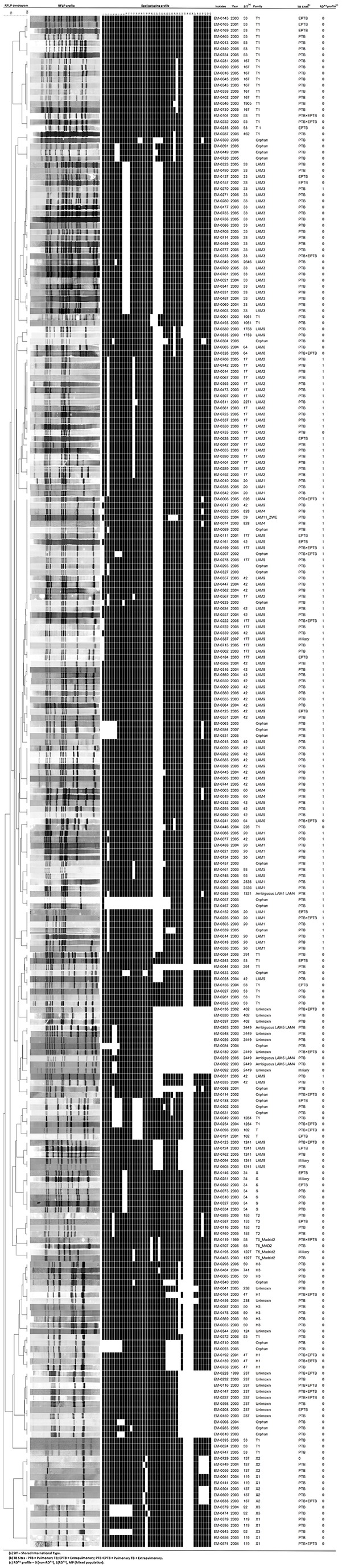
*IS6110* RFLP analysis demonstrated 73 Clustered isolates, Espírito Santo State, Brazil.

The *IS6110* RFLP analysis demonstrated that 250 (41%) isolates belonged to cluster RFLP patterns. Clustered isolates were distributed into 73 groups (Figure 2). Of these clusters, 35 (48%) were comprised of two isolates per cluster, 35 (48%) by 3 to 9 isolates/cluster and 3 (4%) by more than 9 isolates/cluster.

The major RFLP cluster was a 14- band strain comprised of 21 isolates. The ES-14 cluster had 16 LAM - strains and 5 orphan strains; all patients in the cluster had PTB. The second largest cluster included 15 strains from only the LAM family; all patients had PTB. The third largest cluster included 10 LAM family strains; all patients had PTB (Figure 2).

**Figure 3:**
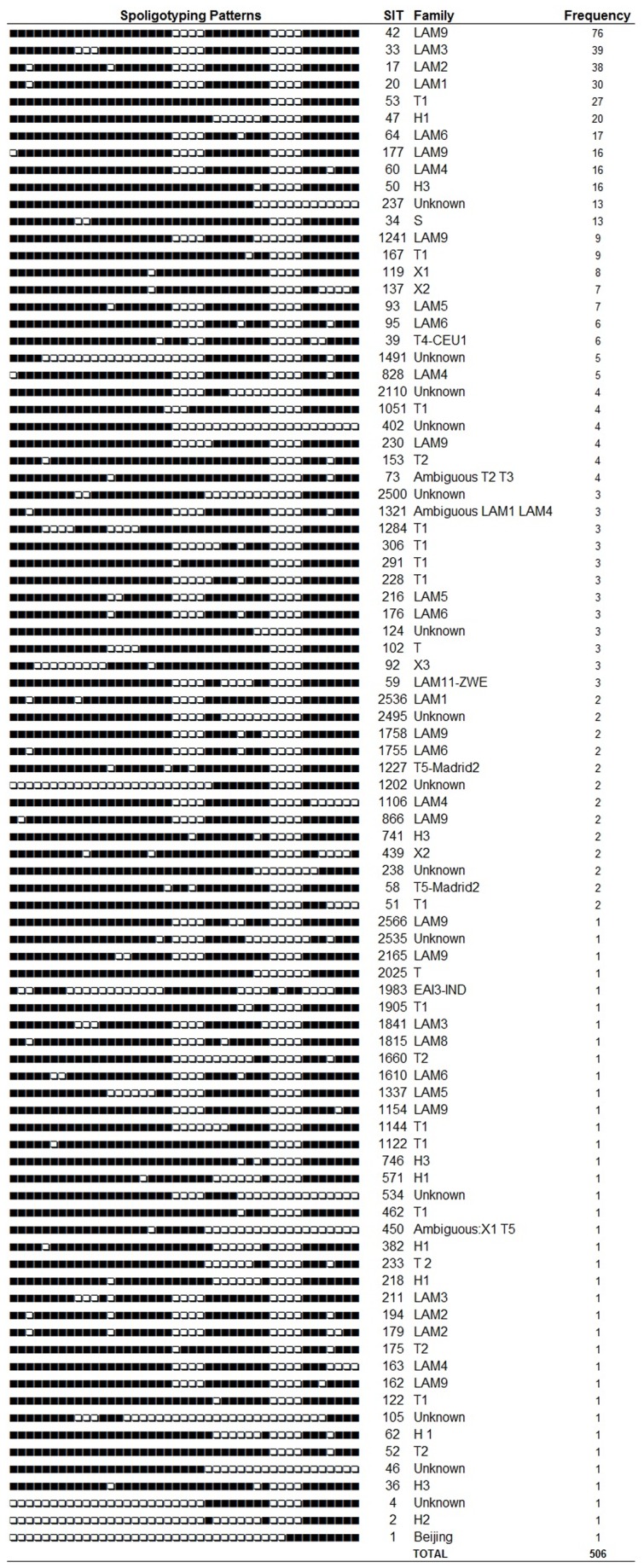
Frequency of Shared International Type of *Mycobacterium tuberculosis* isolates in Espirito Santo State, Brazil.

Spoligotyping data were obtained from 612 isolates: 506 (83%) could be defined to the SIT (Shared International Type) level while 106 (17%) had orphan patterns. Spoligotyping family and SIT information are shown in Figure 3. Among major spoligotyping families, 297 (48%) belonged to the LAM family, 82 (13%) belonged to the T family and 45 (7%) belonged to the Haarlem family.

### PTB and EPTB Characteristics

Characteristics of the study population are shown in Table 2. The median age was 35.9 years (range 2 to 86 years). The majority in both groups were male (63% of EPTB group and 69% of PTB group). Most (49%) were of non-white ethnicity; the proportion of non-whites was lower among those with EPTB (35%) compared to those presenting with PTB (56%). Few patients had more than 7 years of formal education and 96 (16%) were illiterate. Table 2 shows a lower percentage of EPTB cases with AFB smear positive test results (17%) when compared to PTB (79%).

**Table 2:** Demographic, clinical, and molecular microbiologic characteristics, by extrapulmonary TB (EPTB), pulmonary TB (PTB) and both (PTB+EPTB), Espirito Santo, state, Brazil, 1998-2007.

**Characteristics**	**EPTB**	**PTB**	**PTB+EPTB**	**Total**
	**N = 79**	**N = 464**	**N=63**	**N=606**
**Sociodemographic**				
Age (years)				
0 - 9	1 (1%)	4 (1%)	0 (0%)	5 (1%)
10-19	4 (5%)	21 (4%)	1 (1%)	26 (4%)
20-39	44 (56%)	273 (59%)	36 (57%)	353 (58%)
40-60	25 (32%)	143 (31%)	25 (39%)	193 (32%)
> 60	5 (6%)	23 (5%)	1 (1%)	29 (5%)
Gender				
Male	50 (63%)	320 (69%)	54 (86%)	424 (70%)
Female	29 (37%)	144 (31%)	09 (14%)	182 (30%)
Ethnicity				
White	21 (27%)	101 (22%)	7 (11%)	129 (21%)
Non-white	25 (35%)	258 (56%)	16 (26%)	299 (49%)
Not reported	33 (38%)	105 (23%)	40 (63%)	178 (30%)
School Level				
illiterate	22 (28%)	56 (12%)	18 (29%)	96 (16%)
1-3 years	12 (15%)	82 (18%)	7 (11%)	101 (7%)
4-7 years	22 (28%)	123 (26%)	11 (17%)	156 (26%)
>8 years	9 (11%)	91 (20%)	7 (11%)	107 (18%)
Not reported	14 (18%)	112 (24%)	20 (32%)	146 (24%)
**Clinical**				
HIV				
Positive	21 (27%)	20 (4%)	23 (37%)	64 (11%)
Negative	43 (54%)	366 (21%)	33 (52%)	442 (73%)
Not done	15 (19%)	78 (17%)	7 (11%)	100 (16%)
AFB Smear				
Positive	14 (17%)	366 (79%)	20 (31%)	400 (66%)
Negative	62 (79%)	98 (21%)	42 (67%)	202 (33%)
Not done	3 (4%)	0 (0%)	01(02%)	04 (01%)
**Molecular**				
in cluster - RFLP				
Yes	27 (24%)	196 (42%)	29 (46%)	252 (41%)
No	52 (66%)	268 (58%)	34 (54%)	354 (59%)
Rd-Rio				
Present	20 (25%)	147 (32%)	14 (22%)	181 (30%)
Absent	45 (57%)	245 (53%)	39 (62%)	329 (54%)
Not reported	14 (18%)	72 (16%)	10 (16%)	96 (16%)
Spoligotying				
LAM	35 (44%)	234 (50%)	24 (38%)	293 (48%)
Non-LAM	44 (56%)	230 (50%)	39 62%	315 (52%)

HIV: Human Immunodeficiency Virus. RFLP: Restriction Fragment Length Polymorphism. LAM: Latin-American-Mediterranean. AFB; acid fast bacillus. PTB: Pulmonary Tuberculosis EPTB: Extrapulmonary Tuberculosis. PTB+EPTB: both Pulmonary and Extrapulmonary Tuberculosis

HIV co-infection was strongly associated with EPTB. In a logistic regression, EPTB patients were 16.8 times more likely to be HIV-infected than patients with PTB (Table 3).

**Table 3:** Association between sociodemographic, clinical, MTB strain characteristics and TB form, Espírito Santo, state, Brazil, 1998-2007.

	OR*CI95%	OR **CI95%	OR** CI95%	OR**CI95%
**Characteristics**	**EPTB X PTB**	**EPTB X PTB**	**(PTB+EPTB)**	**(PTB+EPTB)**
			**X PTB**	**X EPTB**
**Sociodemographic**				
Age (years)				
Mean	-	0.99 (0.96-1.03)	0.98 (0.93-1.04)	0.97 (0.90-1.06)
Gender				
Female	0.77 (0.46-1.33)	0.71 (0.27-1.87)	1	1
Male	Reference			
Skin				
White	2.14 (1.08-4.18)	2.54 (1.03-6.25)	1.11 (0.24-5.09)	0.17 (0.02-1.45)
Non white	Reference			
School Level				
Illiterate	Reference			
0-3 years	p:0.001	0.36 (0.07-1.82)	0.39 (0.02-5.49)	8.23 (-)
4-7 years		0.16 (0.34-0.73)	0.22 (0.01-2.53)	4.04 (0.09-175)
> 8 years		0.06 (0.009-0.50)	0.63 (0.05-7.05)	7.97 (-)
**Clínical**				
HIV				
Positive	8.93 (4.20-18.81)	116.83 (5.23-54.18)	29.8 (6.04-147.78)	0.97 (0.14-6.70)
Negative	Reference			
AFB Smear				
Positive	0.07 (0.03-0.13)	0.87 (0.56-1.36)	1.28 (0.70-2.33)	1.13 (0.76-1.69)
Negative		Reference		
**Molecular**				
in cluster - RFLP				
Yes	0.70 (0.41-1.19)	0.57 (0.22-1.43)	0.41 (0.10-1.72)	0.48 (0.09-2.38)
No	Reference			
Rd-Rio				
Present	0.74 (0.39-1.33)	0.91 (0.34-2.74)	0.94 (0.21-4.15)	0.61 (0.08-4.76)
Absent	Reference			
Spoligotype				
LAM	0.78 (0.46-1.29)	1.35 (0.47-3.87)	1.81 (0.43-7.55)	0.41 (0.38-4.50)
Non - LAM	Reference			

HIV: Human Deficiency Virus. RFLP: Restriction Fragment Length Polymorphism. LAM: Latin-American-Mediterranean. AFB: acid fast bacillus. PTB: Pulmonary Tuberculosis EPTB: Extrapulmonary Tuberculosis. PTB: Pulmonary Tuberculosis EPTB: Extrapulmonary Tuberculosis.

PTB+EPTB: both Pulmonary and Extrapulmonary Tuberculosis.

* Crude Odds Ratios by Chi-square test ** Adjusted Odds Ratio by Logistic Regression.

Lastly, in a multivariate logistic regression analysis including all variables, only HIV infection (OR: 16.83 95% CI 5.23-54.18), educational level (4-7 years OR: 0.16 95% CI 0.34-0.73 and > 8 years of education, OR 0.06 95% CI 0.009-0.50) and white ethinicity (OR: 2.54 95% CI 1.03-6.25) were associated with EPTB.

We also analyzed a second model that compared characteristics of PTB+EPTB cases with PTB only, and a third model that compared PTB+EPTB cases with EPTB cases only as shown on Table 3. Among these models, only the HIV infection was significant when PTB+ EPTB versus PTB was compared (OR 29.08 95% CI 6-147) (Table 3).

No association with PTB or EPTB was found in the proportion of patients in clusters (cluster index) by *IS6110*-RFLP method (Table 3) and no lineage was associated with EPTB or any of the subsets of EPTB.

**Figure 4:**
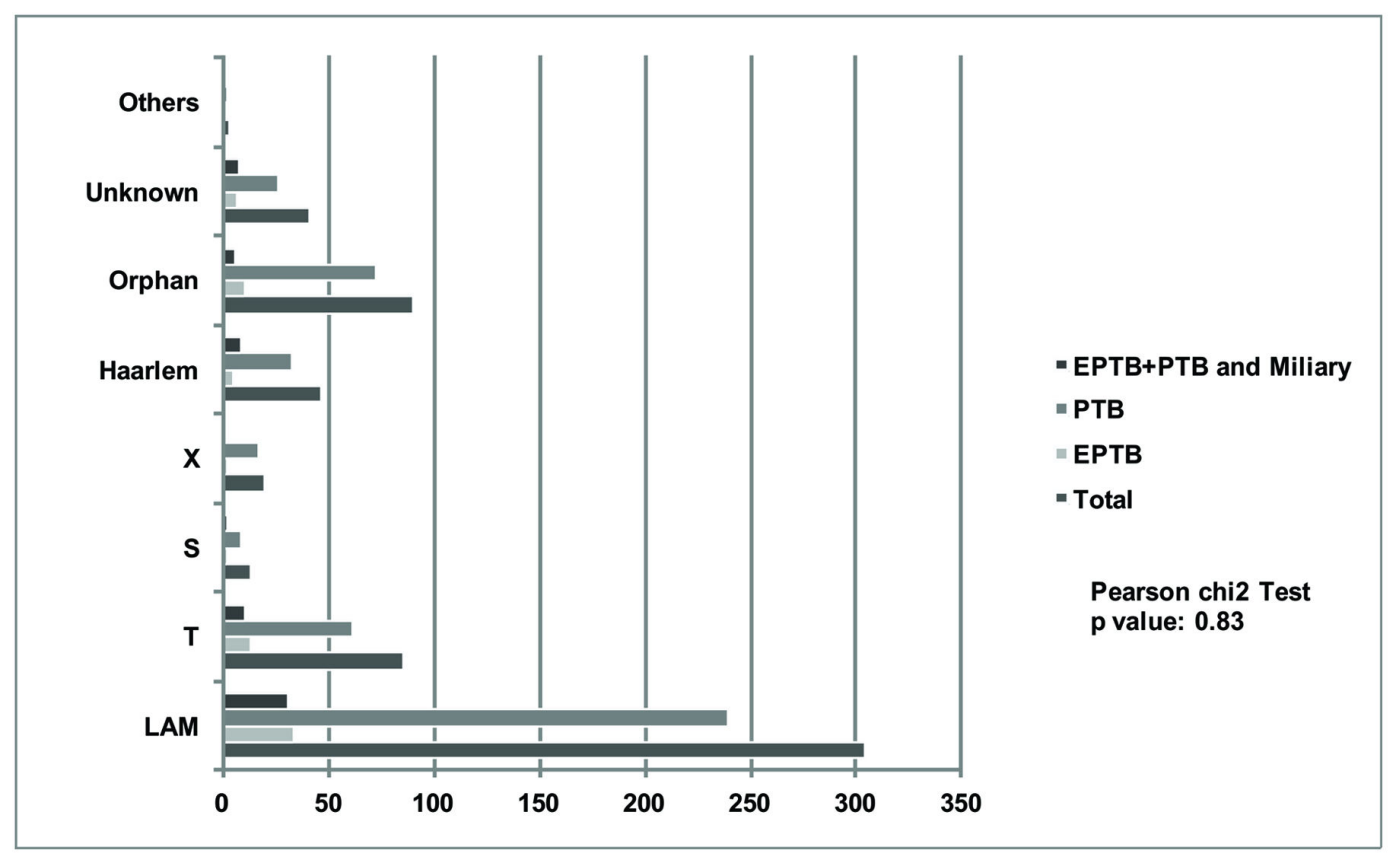
Spoligotyping families by Clinical Site of 606 *M. tuberculosis* isolates, Espirito Santo State, Brazil.

The proportion of patients with PTB and EPTB among spoligotyping families did not differ (p = 0.83, Chi square test) (Figure 4). In Table 3 the categories "LAM" and "non-LAM" were also compared but no differences were found between these groups and clinical form of TB.

The frequency of RDRio sublineage was the last variable that was examined. The PTB and EPTB patients had a similar proportion of people infected with this sublineage (Table 3).

Of 612 isolates, drug susceptibility testing (DST) to first line anti-TB drugs was done for 218 (36%) isolates. Among these, 193 (89%) were from patients with PTB, 18 (8.3%) were from patients with EPTB, 6 (2.8%) were from patients with PTB+EPTB and 1 (0.4%) was from a patient with miliary TB. Isolates from patients with PTB were more likely to undergo DST than those from patients with EPTB (p = 0.0015, Chi-square test). Comparing DST results from patients with PTB and EPTB, 12/181 (6.6%) isolates from patients with PTB were resistant to at least one drug, while 2/18 (11.1%) isolates from patients with EPTB were resistant to at least on drug (p = 0.42, Chi-square test).

## Discussion

This study was conducted to identify factors associated with EPTB in a Brazilian state endemic for TB. In ES state, we found that patients presenting with EPTB were significantly more likely to be of white ethnicity, have a higher educational level, and be infected with HIV. None of the strain genotypic characteristics tested (LAM lineage, RDRio sublineage and RFLP clustering representing recentness of infection) were associated with an increased risk for EPTB.

Our study has several limitations. First, the study was based on culture-positive patients only; mycobacterial cultures are not done for all TB suspects in Brazil, and our database included only 11% of the patients with TB diagnosed in metropolitan area of ES State during the study period. As we used all sample available in the reference laboratory on the data analysis we tried to verify if the non-significance results we found in the association between genotypes and TB outcomes was due to a true lack of relationship or low statistical power (less than 80%). The power based on the sample size we have should be able to detect differences equal to or more than 15%. Therefore, the differences between groups that are less than this could lead to a type 2 error [30]. Second, some epidemiologic data were missing from the national secondary (SINAN) database for some patients. In addition, the low incidence of EPTB reported in national registry may result from underdiagnosis of EPTB due to atypical symptoms, low clinical suspicion, and the recognized difficulty in diagnosing such cases.

Strengths of our study include the analysis of a large number of isolates from a state TB reference laboratory, the similarity of characteristics of the unreported data between patients with and without MTB isolation, the performance of genotyping assays in a single quality controlled laboratory, and the use of three genotyping methods.

The 13% prevalence of EPTB at diagnosis found in this study is in agreement with the 14% overall prevalence reported in Brazil [31]. The most common sites of EPTB were the lymph nodes and pleura, similar to other studies [4,32].

In contrast to other studies [6,12,33], we observed a significantly higher frequency of EPTB among whites than non-whites in ES State. TB more frequently affects non-whites in Brazil [34]. The more frequent presentation of EPTB in whites agrees with another recent national study in Brazil that also found a significant association between white ethnicity and EPTB [34]. Forty-five per cent of patients with EPTB were of white ethnicity in that study [35]. We note, however, that in SINAN, ethinicity is based on self-reported data and that in Brazil, where a large proportion of the people are of mixed ethnicity, there is likely to be some misclassification.

We also found that EPTB and PTB were more common in males than in females, but in the literature EPTB has been reported more frequently in women [5].

Our analysis showed that a higher school level was associated with EPTB, which is in contrast to to other reports of low educational level and TB in general. In a study in Ribeirão Preto, SP, TB rate was correlated with lower educational level and social vulnerability [36]. This association with a higher level education follows the characteristic of EPTB in Brazil, where 28%, a significant percentage of the 53,853 EPTB patients have five to eight years of education [35] Most patients with EPTB had negative sputum smears which is expected as number of bacteria in extrapulmonary specimens is usually smaller than the number in lung specimens. Furthermore, the extrapulmonary lung collection of materials often requires invasive procedures and it is difficult to obtain additional samples [37].

HIV co-infection was the main factor associated with EPTB. The association of HIV infection and EPTB is expected, since EPTB occurrence increases in frequency when cellular immune function is compromised [38,39]. Also HIV is a known risk factor for progression of *M. tuberculosis* infection to active disease, increasing the risk by 20-fold [40]. One limitation of the SINAN database was the absence of individual CD4 count, and more complete and comprehensive national reporting of cases of TB in HIV-infected persons are needed. The high number of HIV-positive patients in our study population may have been influence by the fact that MTB culture is not universal in Brazil and HIV-infected people are included among vulnerable populations to be cultured when suspected to have TB [17]. We found no difference in the frequency of drug resistant TB comparing patients with PTB and EPTB.

Based on RFLP cluster pattern analysis we found no difference between recent infection or reactivation disease according to clinical presentation with PTB or EPTB. A similar observation was made in a study from Madagascar among 316 isolates from patients presenting with different clinical sites of TB [41]. In a US study that examined 5,085 EPTB cases, patients with EPTB were more likely to be infected with Euro-American (adjusted OR, 1.3; 95% CI, 1.1-1.4), Indo-Oceanic (adjusted OR, 1.7; 95% CI, 1.5-1.9), and East-African Indian (adjusted OR, 1.6; 95% CI, 1.4-1.9) lineages [12]. The difference in results between our study and the US study could be related to the difference in size of the study population and low variability of the population susceptible to TB in Brazil (e.g., most patients in our study are Brazil-born, as opposed to US patients with TB, many of whom are immigrants), or that there is indeed no strong relationship between MTB lineage and EPTB, as was reported also from India [13]. The fact that we were able to show several host-related factors to be associated with EPTB suggests that even if MTB strain-related factors play a role in this association, such factors may not be major contributors, as also indicated by the small ORs reported from the US study.

RDRio was previously suggested to show enhanced virulence so it was analyzed for its association with EPTB [42]. We did not find a statistically significant association between the RDRio lineage and EPTB. A recent study from our group showed that it is not clear if this high prevalence of RDRio is attributed to its enhanced intrinsic virulence or high transmissibility [25].

A result that deserves attention was the large proportion of EPTB cases in clusters, since clusters are suggestive of recent transmission [43]. The association of pleural TB with recent transmission chains has been reported, with the idea that pleural TB is an early sign of primary infection by MTB, and also can be detected earlier than other forms of PTB and EPTB [44]. However, in our study, the proportion of MTB belonging to *IS6110* RFLP clusters in pleural TB (30.6%) was not significantly different from all forms of EPTB (39.2%). These results suggest that risk factors for EPTB are related more to host factors than to MTB strain lineage characteristics.

The large proportion of clustered MTB strains in both PTB and EPTB cases highlights the high recent transmission rates currently occurring in ES State. This study suggests that strengthening efforts to interrupt new transmission should have a similar impact on the control of both PTB and EPTB.
